# Predictive value of hyponatremia for short-term mortality in supratentorial spontaneous intracerebral hemorrhage: a single center study

**DOI:** 10.3389/fneur.2024.1301197

**Published:** 2024-01-25

**Authors:** Ao Qian, Longyi Zheng, Zeyuan He, Jing Zhou, Shuang Tang, Wenli Xing

**Affiliations:** ^1^Department of Cerebrovascular Disease, Suining Central Hospital, Suining, Sichuan, China; ^2^Department of Radiology, Xiang’an Hospital of Xiamen University, School of Medicine, Xiamen University, Xiamen, China; ^3^Department of Neurosurgery, The First Affiliated Hospital of Chongqing Medical University, Chongqing, China

**Keywords:** intracranial hemorrhage, hyponatremia, 90-day, mortality, functional outcome

## Abstract

**Background:**

Hyponatremia is a common electrolyte disturbance in patients with neurological disease; however, its predictive role for outcome in patients with supratentorial spontaneous intracerebral hemorrhage (sICH) is controversial. This study aims to explore the association between hyponatremia within 7 days after bleeding and 90-day mortality in patients with supratentorial sICH.

**Methods:**

A retrospective analysis was conducted at our institution. Patients with sICH meeting the inclusion criteria were enrolled in this study. Multivariate regression analyses were performed to determine the predictive value of hyponatremia (serum sodium <135 mmol/L) for 90-day mortality and functional outcome. Subgroup analysis was performed based on the degree and duration of hyponatremia and therapeutic strategies. The Spearman correlation test was performed to explore the relationship between hyponatremia severity and duration with variables in a multivariate regression model. Kaplan–Meier curve was depicted to reveal the relationship between hyponatremia and mortality. The receiver operating characteristic (ROC) curve was plotted to show the diagnostic effect of the minimum concentration of serum sodium (sodium_min_) on 90-day mortality.

**Results:**

A total of 960 patients were enrolled, 19.6% (188) of whom were patients with hyponatremia and 26.0% (250) had 90-day mortality. The incidence of hyponatremia was roughly 2.5 times in non-survivors compared with survivors (34.8% vs. 14.2%). Multivariate regression analysis revealed that hyponatremia was the independent predictor of 90-day mortality (OR 2.763, 95%CI 1.836–4.157) and adverse outcome (OR 3.579, 95%CI 2.332–6.780). Subgroup analysis indicated an increased trend in mortality risk with both duration (more or less than 48 h) and severity of hyponatremia (mild, moderate, and severe) and confirmed the predictive value of hyponatremia for mortality in patients undergoing surgical intervention (external ventricular drainage, craniotomy, and decompressive craniectomy; all *p* < 0.05). The Spearman correlation test indicated no moderate or strong relationship between hyponatremia severity and duration with other variables in the multivariate model (all |r_s_| < 0.4). The ROC curve suggested the moderate diagnostic performance of sodium_min_ for mortality in both general patients and subgroups of therapeutic method patients (AUC from 0.6475 to 0.7384).

**Conclusion:**

Hyponatremia occurring in the first 7 days after bleeding is an independent predictor of 90-day morality and adverse outcome. Rigorous electrolyte scrutiny in patients treated surgically is required.

## 1 Introduction

Hyponatremia is the most frequent electrolyte disturbance in hospitalized patients, serving as a predictor of adverse outcomes in various diseases, such as heart failure, cirrhosis, and chronic kidney disease (1–4). In critical neurological conditions, hyponatremia after subarachnoid hemorrhage (SAH) and traumatic brain injury also indicate poor prognosis or increased duration of hospital stay (5, 6). Syndrome of inappropriate antidiuretic hormone (SIADH) and cerebral salt wasting syndrome (CSWS) may be the main etiologies of decreased serum sodium in neurological injury, and the pathophysiological mechanism of hyponatremia linking to adverse outcome includes exacerbated cerebral edema and seizures (7–9).

Spontaneous intracerebral hemorrhage (sICH) accounts for approximately 10–30% of stroke and is considered the most devastating form of this disease, with a high rate of mortality and morbidity (10, 11). Numerous factors predicting poor prognosis have been put forward, including age, Glasgow Coma Scale (GCS) score at admission, hematoma location (infratentorial), hemorrhage volume, and intraventricular hemorrhage (IVH) (12–14). However, the association of decreased serum sodium with the prognosis of patients with sICH is still undetermined. Until now, only three studies have identified the predictive role of hyponatremia in mortality of ICH in detail (15–20). However, all of these studies lack integrated analysis in considering the different levels of severity (mild, moderate, and severe) of hyponatremia. In addition, enrolled patients without a definite occurrent time of hyponatremia may impair the consistency of the cohort. Therefore, we conducted a retrospective study at our institution with the primary purpose of identifying the predictive value of hyponatremia in the early stage (within 7 days) of supratentorial sICH for 90-day mortality. Subgroup analysis was also performed according to duration and degree of hyponatremia, as well as different therapeutic strategies. Our secondary objective was to explore the association between hyponatremia and 90-day functional outcomes in patients with sICH.

## 2 Methods

### 2.1 Study design

This study was approved by our institutional ethics committee (approve number: KYLLMC20230022). Data for patients with supratentorial sICH were retrospectively collected at Suining Central Hospital from January 2019 to April 2023. Management and surgical treatment were performed by the same group of senior surgeons. Date points, including demographics, previous medical history, imaging findings, admission GCS sore and ICH score, serological tests, complications, and follow-up information, were reviewed. Patient selection was based on the following inclusion criteria: (1) patients with hemorrhage in the supratentorial location; (2) patients admitted to our institution within 24 h after hemorrhage onset; (3) patients who had serum monitoring at least 3 times (each interval ≥ 24 h) within 7 days after bleeding. The excluding criteria were as follows: (1) secondary intracerebral hemorrhage related to trauma, tumor, arteriovenous malformations, coagulopathy, or hemorrhagic transformation of ischemic stroke; (2) hemorrhage caused by antiplatelet or anticoagulant drugs; (3) patients with other decompensated underlying diseases or progressive comorbidities (such as renal, liver, or cardiac disease) before ICH onset; (4) patients with a history of stroke had residual dysfunction before bleeding; (5) patients without sufficient follow-up data; (6) patients with age less than 18 years old. A comprehensive emergency assessment was performed for each patient after admission at an emergency room within 30 min, including a GCS score for consciousness, computed tomography (CT) scan for hemorrhage volume and IVH, and hematologic examinations for peripheral blood cells, hepatorenal function, electrolyte, and coagulation function. Then, the patients would be transferred to the general ward, the neurological intensive care unit of our department, or the operating room. In addition, CT angiography for conservatively treated patients or digital subtraction angiography for surgically treated patients was also routinely employed to rule out certain cerebrovascular diseases (21). Parenchymal volume was calculated using the ABC/2 formula (A was the largest diameter on the widest hemorrhage slice; B was the diameter perpendicular to A; C was the thickness of hemorrhage obtained by axial CT slice) (22, 23). The extent of IVH was evaluated using the Graeb score (24).

Hyponatremia was defined as serum sodium concentration < 135 mmol/L and rated as three grades according to the minimum concentration of serum sodium (sodium_min_): mild (130–134 mmol/L), moderate (125–129 mmol/L), and severe (< 125 mmol/L) (25). Critically, in this study, we only review the records of sodium in the first 7 days after bleeding to maintain consistency throughout the cohort. Outcome measures (mortality and functional outcome) were evaluated 90 days after hemorrhage onset using the modified Rankin Scale (mRS) by outpatient or phone interview. The mRS scores were dichotomized to assess favorable and adverse outcomes (mRS 0–3 versus 4–6) (17).

### 2.2 Protocol for hyponatremia

Our protocol for sodium monitoring was as follows:

1. Routinely, serum sodium was measured daily for the first 3 days after bleeding and every 2 days thereafter until discharge.
2. For patients with hyponatremia, 0.9% sodium infusions or 3% hypertonic saline infusions were applied according to daily monitoring of electrolyte, hematocrit, hemoglobin, and serum albumin until values of serum sodium became normal. To maintain the stability of hyponatremia correction, oral sodium was not applied for hyponatremia correction.
3. The process of hyponatremia correction should be precisely controlled with sodium fluctuation ≤8 mmol/L per 24 h.

In addition, we also attempt to perform *post hoc* evaluation of hyponatremia etiology (SIADH, CSWS, or other) by reviewing medical records for signs of hypovolemia, laboratory evidence of dehydration (hematocrit, hemoglobin, serum albumin, or blood urea), fluid balance, and central venous pressure (if available). The diagnosis of SIADH or CSWS was in accordance with the criteria reported in previous studies (9, 15, 26). Briefly, the diagnosis of CSWS depended on the presence of at least two features meeting the following conditions.

1. Manifestation of hypovolemia: dry mucous membranes, hypotension, or tachycardia.
2. Negative fluid balance of more than 1,000 mL.
3. Evidence of dehydration: elevated hemoglobin, hematocrit, blood urea, or serum albumin.
4. Central venous pressure (CVP) < 6 cm of water.

The diagnosis of SIADH depended on the presence of at least two features meeting the following conditions.

1. No sign of hypovolemia.
2. Normal of positive fluid balance.
3. No sign of dehydration: normal or decreased hemoglobin, hematocrit, blood urea, or serum albumin.
4. CVP > 6 cm of water.

### 2.3 Treatment strategies

The treatment strategy was individualized according to the patient’s condition, including conservative therapy and surgical intervention. All surgical procedures were performed within 24 h after sICH onset with the consent of relatives, including external ventricular drainage (EVD), craniotomy of hematoma evacuation, and decompressive craniectomy (DC) in addition to hematoma removal. EVD was indicated by massive hematoma with prominently intraventricular extension, with the existence or trend of hydrocephalus (13). The indications for craniotomy were hemorrhage volume > 30 mL with a midline shift >0.5 cm, and an obvious lateral ventricle compression (27). Decompressive craniectomy would be performed if intracerebral pressure was expected to significantly increase after hematoma evacuation due to preoperative large ICH volume and severe midline displacement (28). The specific procedures of these three surgical methods have been reported in previous studies (29, 30). The time period from hemorrhage to surgical intervention was recorded to explore its impact on hyponatremia. The peri-operative and conservative management contained comprehensive neurologic care (prevention of venous thrombus embolism, maintenance of nutrition, etc.), appropriate drug treatment (osmotic agents, hemostatic, hypotensor, antibiotics if infection occurred, etc.), rigorous serological monitoring (peripheral blood cells, hepatorenal function, myocardial enzyme, electrolyte, coagulation function, etc.), and maintenance of fluid balance (13, 15).

### 2.4 Statistical analysis

Variables in univariate analysis were selected based on our experience and previous studies (12, 14, 20). The outcome of 90-day mortality and mRS score were also explored in previous studies of stroke (12, 31, 32). Continuous variables were presented as mean ± SD (compared using Student’s *t*-test) or median with interquartile range (compared using Mann–Whitney *U*-test) as appropriate after normality test. Categorical variables were presented as a percentage with comparison using the Chi-square or Fisher’s exact test. Parameters reaching statistical significance (*p* < 0.05) were enrolled into multivariate logistic regression models to identify the predictive factors of 90-day mortality and adverse outcomes. Three subgroups were classified based on the severity of hyponatremia (mild, moderate, and severe), as mentioned previously. In addition, cases with hyponatremia were also categorized into another two subgroups based on the duration: < 48 h and > 48 h. Three degrees of hyponatremia or two groups of hyponatremia duration were enrolled in multivariate models with the non-hyponatremia as the reference group to further assess the predictive value of hyponatremia for 90-day mortality. Subgroup analysis was also performed to explore the effect of hyponatremia in patients with different therapeutic strategies (conservative, EVD, craniotomy, and decompressive craniectomy). The presence of multicollinearity among independent variables was assessed using weighted linear regression, defined as variance inflation factor ≥ 5. The Spearman correlation test was performed to explore the relationship between hyponatremia severity and duration with variables in the multivariate regression model. Kaplan–Meier curve was depicted to reveal the relationship between hyponatremia and mortality. The receiver operating characteristic (ROC) curve was plotted to show the diagnostic effect of sodium_min_ on 90-day mortality. All statistical analyses were carried out using SPSS 25.0, and *p* < 0.05 was considered statistically significant.

## 3 Results

### 3.1 Demographics

**Figure 1:**
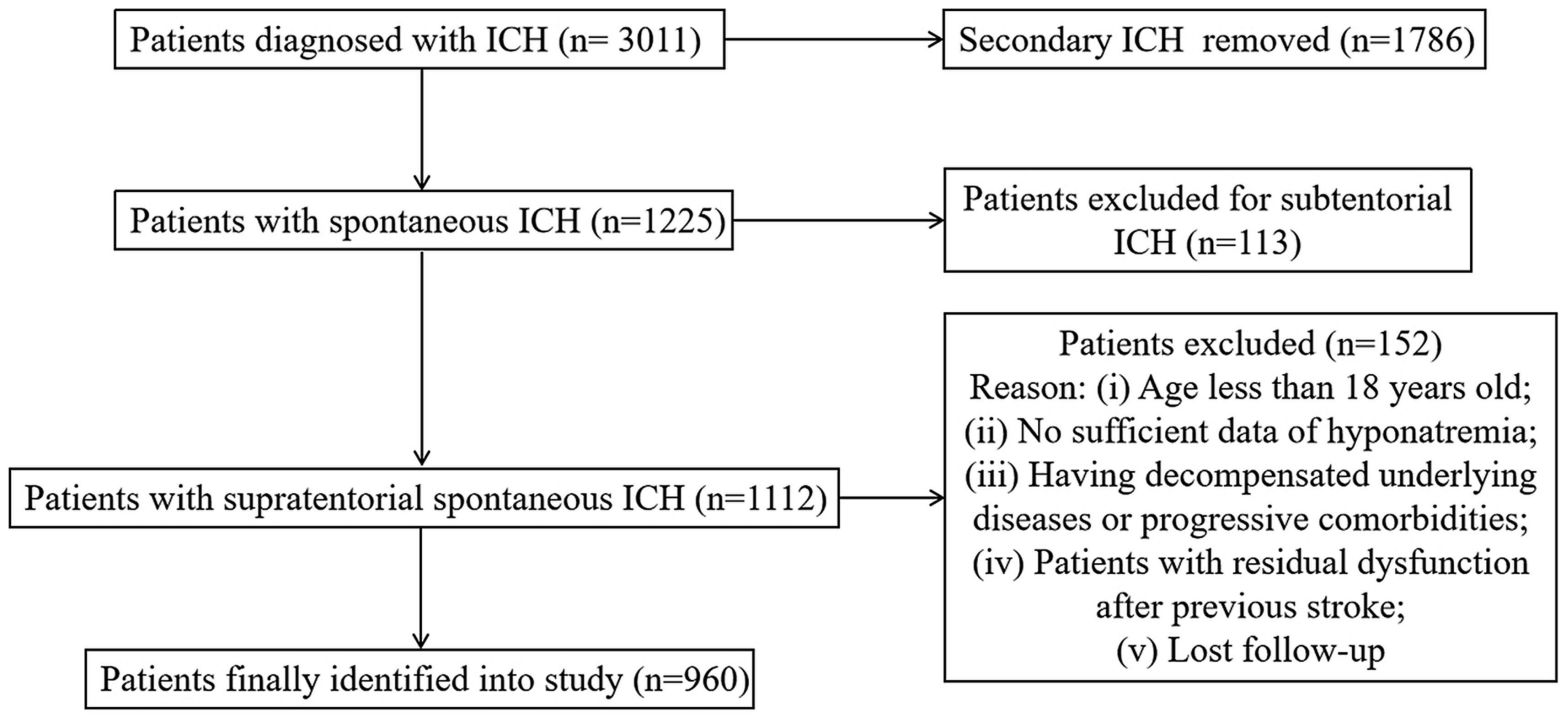
The flow diagram of patient selection.

A total of 960 patients were enrolled in this study, and the process of patient selection is shown in Figure 1. The incidence of hyponatremia was 19.6% (n = 188), with mild, moderate, and severe severity rates of 8.7% (84), 6.3% (61), and 4.4% (46), respectively. For the duration of hyponatremia, 11.8% (114) of patients were normalized in less than 48 h, while 7.7% (74) of patients were normalized in over 48 h. None combined with renal or liver disorder during the duration of hyponatremia. Speculatively, in cases of hyponatremia, 122 (64.9%) were caused by SIADH, 42 (22.3%) had CSWS, and 24 (12.8%) may have been caused by miscellaneous factors. At the 90-day outcome assessment, 250 (26.0%) patients had died and 554 (57.7%) patients had adverse outcomes (mRS = 4 to 6). As for therapy, 588 (61.3%) patients were treated with the conservative method, and 372 (38.7%) patients underwent surgical intervention, including EVD for 61 patients (6.4%), craniotomy for 170 patients (17.7%), and DC for 141 (14.7%) patients. The details of patients who underwent different therapeutic strategies are summarized in Table 1. The median time period from hemorrhage to surgical intervention was 4 h (1–22 h), 205 cases at ≤4 h and 167 cases at >4 h. A similar incidence of hyponatremia was found in these two groups (≤ 4 h vs. > 4 h = 28.3% vs. 25.1%, *p* = 0.496).

**Table 1:** Hyponatremia and outcomes of patients under different treatment strategies.

	Total	Conservative	EVD	Craniotomy	DC
Patients	960	588	61	170	141
Hyponatremia	188	88	19	40	41
Death	250	121	21	59	49
Functional outcome					
mRS 0–3	406	284	19	66	37
mRS 4–6	554	304	42	104	104

EVD, external ventricular drainage; DC, decompressive craniectomy; mRS, modified rankin scale.

### 3.2 Hyponatremia is independently associated with 90-day mortality

**Figure 2:**
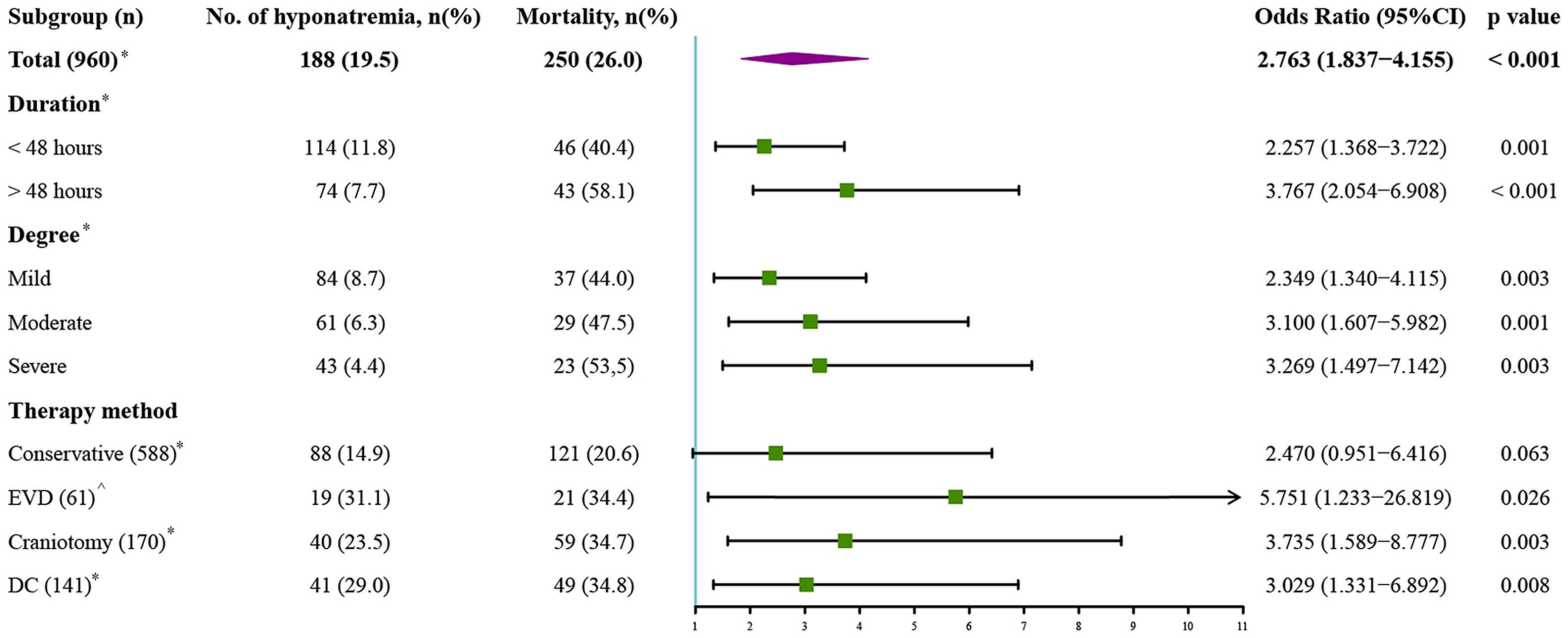
Subgroup analysis to explore the predictive value of hyponatremia in different conditions. EVD, external ventricular drainage; DC, decompressive craniectomy. *Model adjusted for age, Graeb score, hemorrhage volume, diabetes mellitus, GCS score, and ICH score. ^Model adjusted for age, Graeb score, diabetes mellitus, GCS score, and ICH score.

**Figure 3:**
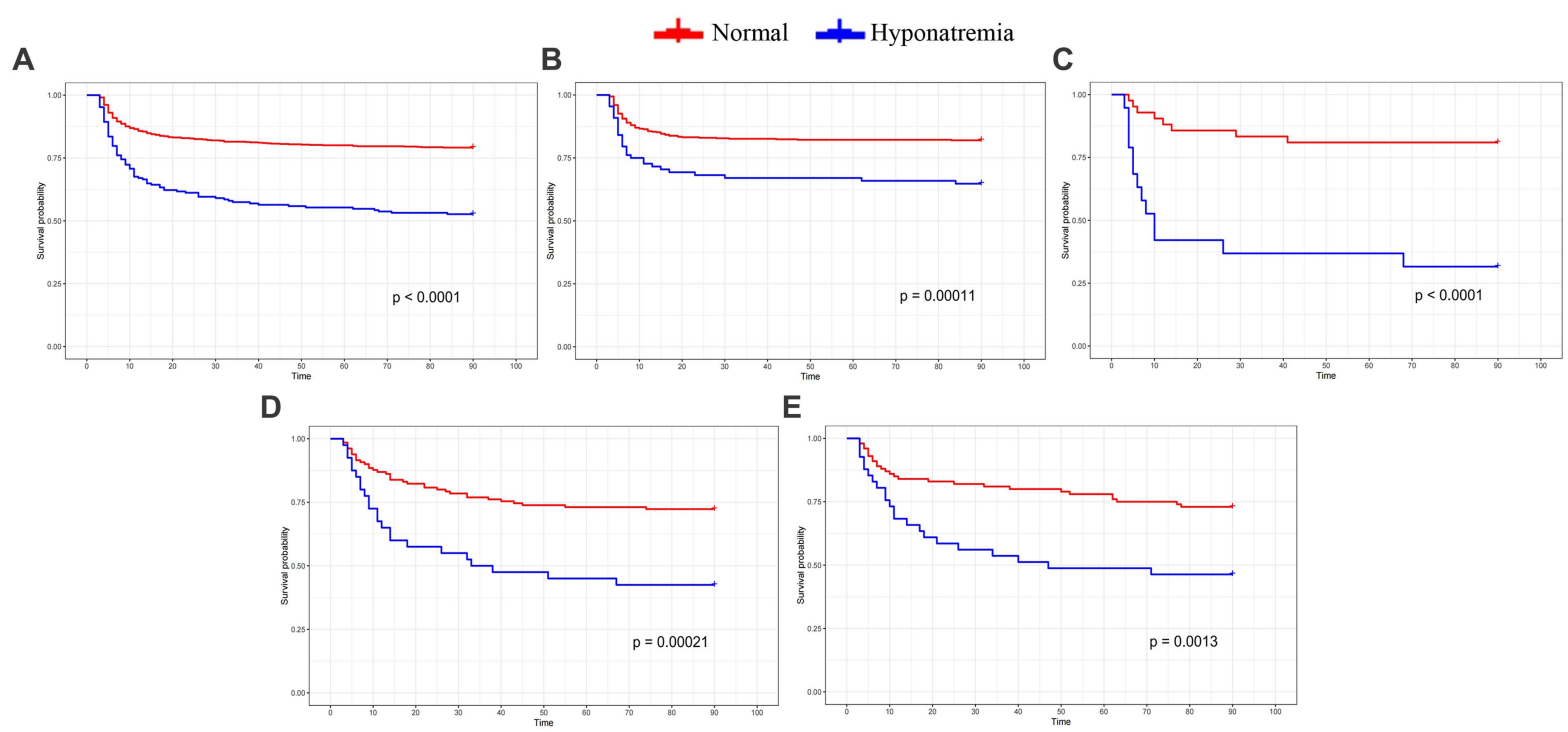
Kaplan-Meier curve indicates the relationship between hyponatremia and mortality in the whole cohort and subgroups with different therapeutic strategies. (A) the whole cohort. (B) Conservative treatment group. (C) EVD group. (D) Craniotomy group. (E) DC group.

**Figure 4:**
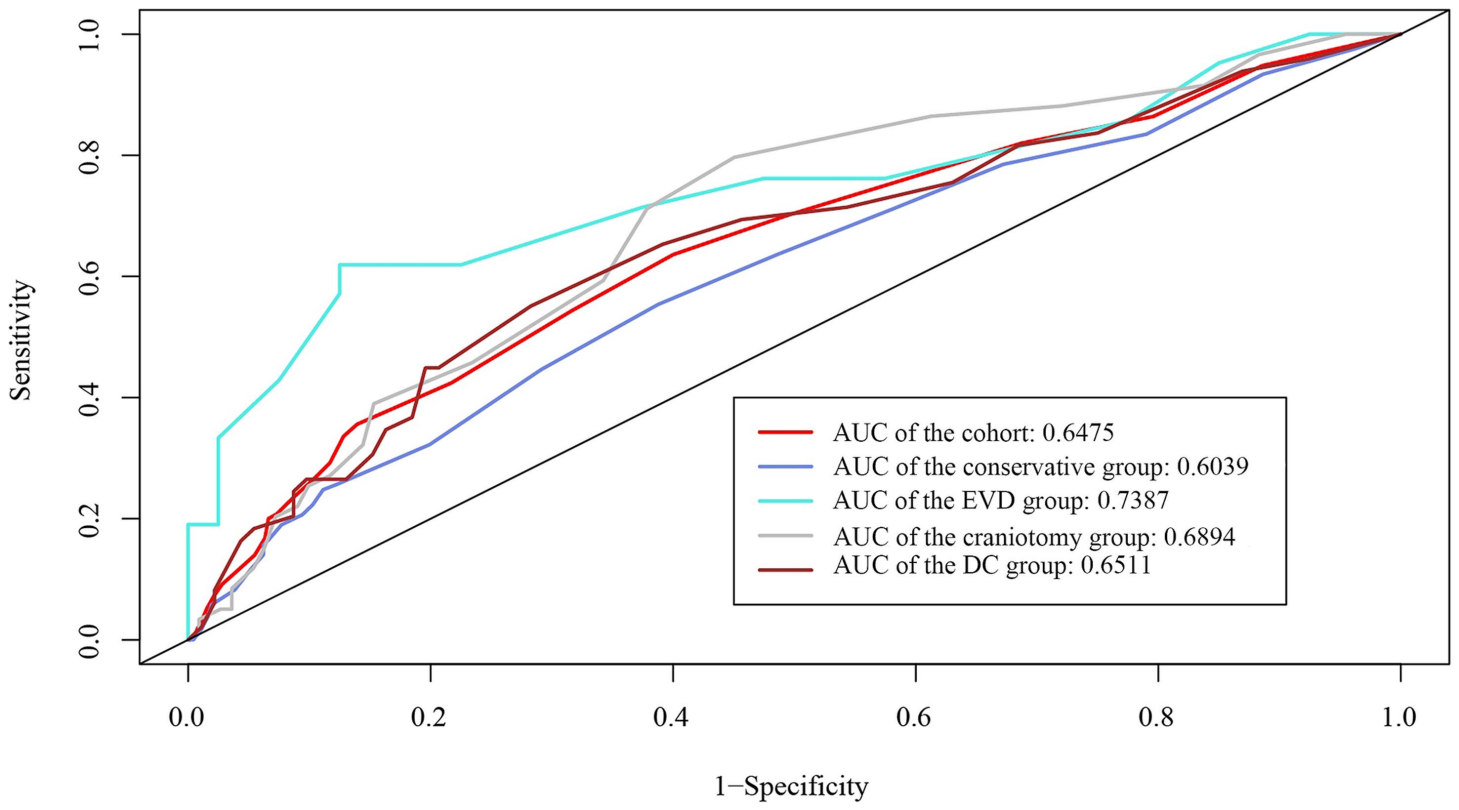
ROC curve demonstrates the moderate diagnostic value of sodium_min_ in mortality in the whole cohort and subgroups with different therapeutic strategies.

**Figure 5:**
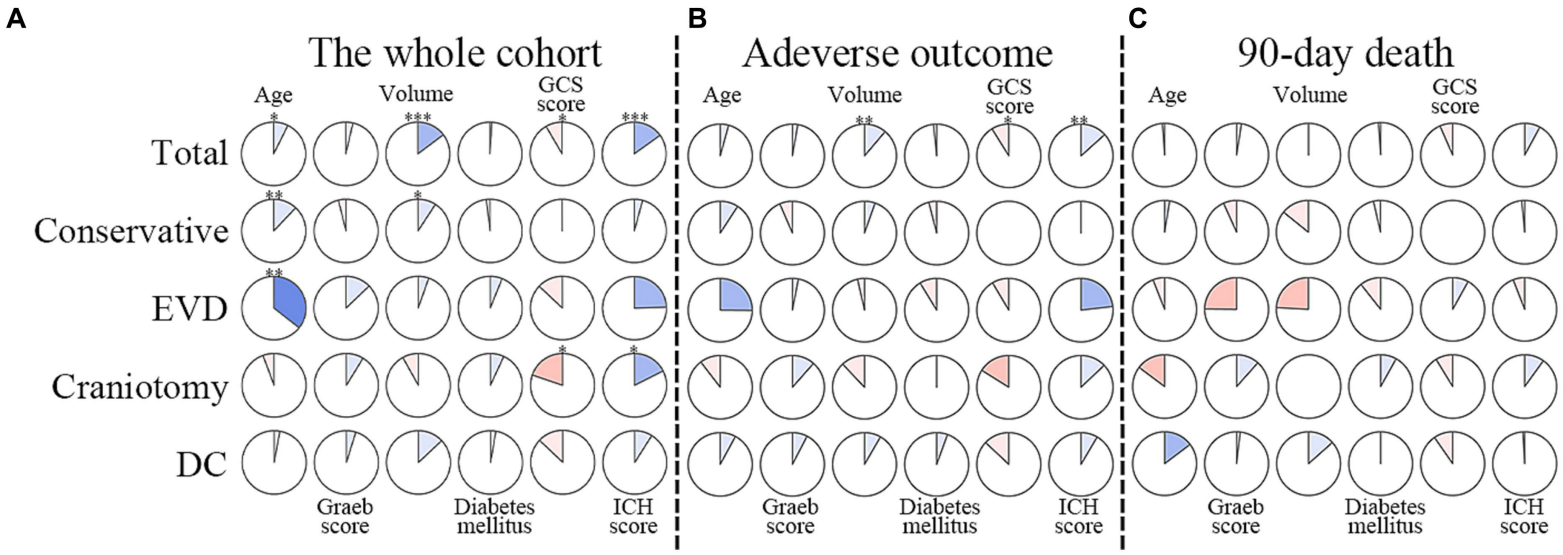
Correlations between degrees of hyponatremia and factors in multivariable models. The clockwise and counterclockwise graphs demonstrate positive and negative correlations, respectively. *, **, and *** indicate *p* < 0.05, *p* < 0.01, and *p* < 0.001, respectively. **(A)** Presents the correlations in the whole cohort. **(B)** The group with adverse outcomes. **(C)** Shows patients who had died by the 90-day follow-up. EVD external ventricular drainage; DC decompressive craniectomy.

**Figure 6:**
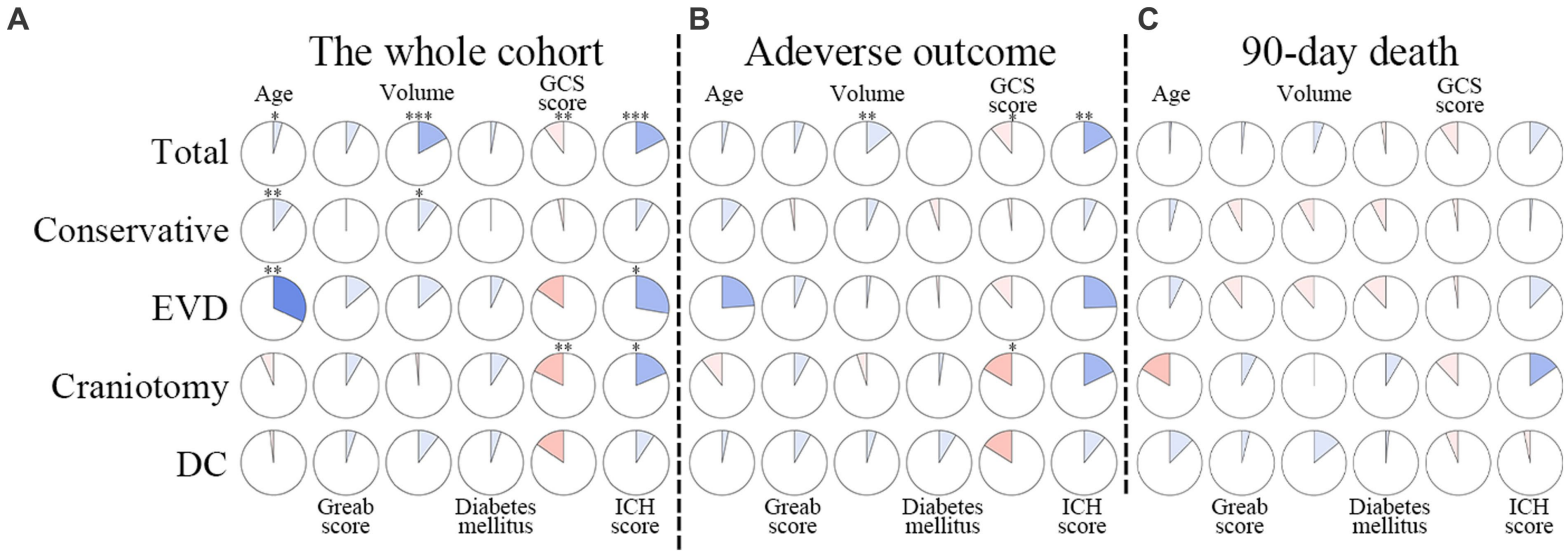
Correlations between duration of hyponatremia and factors in multivariable models. The clockwise and counterclockwise graphs demonstrate positive and negative correlations, respectively. *, **, and *** indicate *p* < 0.05, *p* < 0.01, and *p* < 0.001, respectively. **(A)** Presents the correlations in the whole cohort. **(B)** The group with adverse outcomes. **(C)** Shows patients who had died by the 90-day follow-up. EVD external ventricular drainage; DC decompressive craniectomy.

The whole cohort was categorized into groups of survivors and non-survivors based on the 90-day outcome. Baseline information is summarized in Table 2. Patients in the non-survivor group had a significantly higher rate of hyponatremia than those in the survivor group (34.8% vs. 14.2%, *p* < 0.001). Moreover, the sodium_min_ and the mean concentration of serum sodium (sodium_mean_) within 7 days after bleeding in the non-survivor group were also significantly lower, compared to those in the survivor group (sodium_min_: 134.8 ± 6.1 vs. 137.7 ± 4.8; sodium_mean_: 138.1 ± 5.4 vs. 140.7 ± 4.4; both p < 0.001). Multivariate analysis showed that hyponatremia was independently associated with mortality (OR 2.763, 95%CI 1.837–4.157, p < 0.001) after adjusting for age, IVH (Graeb score), hemorrhage volume, diabetes mellitus, admission GCS score, and ICH score (Table 3). Furthermore, increased trend of mortality risk with both duration (< 48 h: OR 2.257, 95%CI 1.368–3.722; > 48 h: OR 3.767, 95%CI 2.054–6.908) and severity of hyponatremia (mild: OR 2.349, 95%CI 1.340–4.115; moderate: OR 3.100, 95%CI 1.607–5.982; severe: OR 3.269, 95%CI 1.497–7.142) was found in subgroup analysis (Figure 2). In addition, a predictive role of hyponatremia for mortality was also identified in the subgroups of surgical intervention (EVD: OR 5.751, 95%CI 1.233–26.819; craniotomy: OR 3.735, 95%CI 1.589–8.777; DC: OR 3.029, 95%CI 1.331–6.892; Figure 2). However, the statistical significance of the conservative subgroup was marginal (OR 2.470, 95%CI 0.951–6.416, *p* = 0.063). Kaplan–Meier survival curves from day 0 until day 90 demonstrated the association between higher mortality and hyponatremia in the whole cohort and patients under different therapeutic strategies (all *p* < 0.05, Figure 3). The ROC curve suggested the moderate diagnostic performance of sodium_min_ for mortality in both general patients and subgroups of therapeutic methods (AUC from 0.6475 to 0.7384, Figure 4). Spearman correlation test indicated no moderate or strong relationship between hyponatremia severity and duration with other variables in the multivariate model (all |r_s_| < 0.4). In addition, no significant correlation was found in patients who had died before the 90-day follow-up (all *p* > 0.05) (Figures 5, 6).

**Table 2:** Comparisons of demographics between survivors and non-survivors.

	Survivor	Non-survivor	Value of *p*
Patients	710	250	
Age (years)	64.8 ± 12.2	67.4 ± 12.4	0.004
Male, *n* (%)	454 (63.9)	150 (60.0)	0.267
BMI	25.1 (23.2–27.6)	24.8 (22.7–27.5)	0.460
Hypertension, *n* (%)	574 (80.8)	199 (79.6)	0.669
Diabetes mellitus, *n* (%)	147 (20.7)	68 (27.2)	0.032
Dyslipidemia, *n* (%)	80 (11.3)	38 (15.2)	0.306
Hypercholesterolemia, *n* (%)	179 (25.2)	74 (29.6)	0.176
Atrial fibrillation, *n* (%)	74 (10.4)	29 (11.6)	0.605
Previous stroke, *n* (%)	110 (15.5)	46 (18.4)	0.284
History of CHD, *n* (%)	23 (3.2)	7 (2.8)	0.731
History of renal insufficiency, *n* (%)	78 (11.0)	32 (12.8)	0.439
History of liver disease, *n* (%)	171 (24,1)	56 (22.4)	0.590
Hematoma volume (ml)	17 (13–34)	35 (31–45)	< 0.001
IVH, *n* (%)	343 (48.3)	184 (73.6)	< 0.001
Graeb score	0 (0–2)	2 (0–3)	< 0.001
Hematoma location, *n* (%)			0.298
Deep	610 (85.9)	208 (83.2)	
Lobar	100 (14.1)	42 (16.8)	
Admission GCS score	13 (8–15)	10 (9–13)	< 0.001
Admission ICH score	1 (1–2)	2 (2–3)	< 0.001
Hypernatremia, *n* (%)	113 (15.9)	37 (14.8)	0.676
Hyponatremia, *n* (%)	101 (14.2)	87 (34.8)	< 0.001
Sodium_min_	137.7 ± 4.8	134.8 ± 6.1	< 0.001
Sodium_mean_	140.7 ± 4.4	138.1 ± 5.4	< 0.001

BMI, body mass index; CHD, coronary heart disease; GCS, glasgow coma scale; ICH, intracerebral hemorrhage.

**Table 3:** Multivariable model for predictors of 90-day mortality.

	OR (95%CI)	Value of *p*	VIF
Age	1.021 (1.007,1.036)	0.003	1.015
IVH (Graeb score)	1.169 (1.052,1.299)	0.004	1.549
Hemorrhage volume	1.050 (1.035,1.066)	< 0.001	1.640
Diabetes mellitus	1.329 (0.875,2.019)	0.182	1.004
Admission GCS score	1.079 (1.008,1.154)	0.029	1.665
Admission ICH score	2.432 (1.778,3.327)	< 0.001	3.181
Hyponatremia	2.763 (1.837,4.157)	< 0.001	1.034

IVH, intraventricular hemorrhage; GCS, glasgow coma scale; ICH, intracerebral hemorrhage; VIF, variance inflation factor.

### 3.3 Hyponatremia is a predictor of adverse outcomes

**Figure 7:**
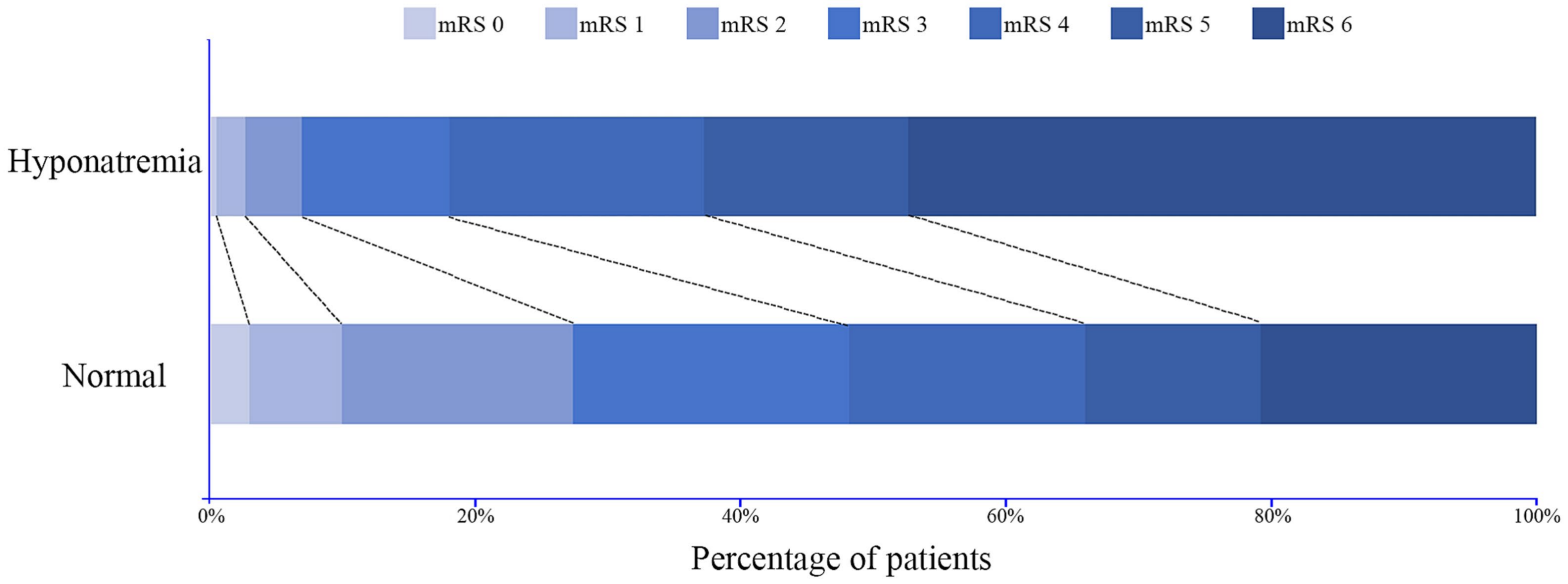
Distribution of the modified Rankin Scale (mRS) score at 90-day follow-up for patients with and without hyponatremia.

The distribution of mRS score in patients with or without hyponatremia is depicted in Figure 7. Patients were divided based on mRS score at the 90-day outcome (favorable and adverse), and the details of these two groups are summarized in Table 4. Similarly, patients with adverse outcomes had a significantly higher rate of hyponatremia and lower sodium_min_ and sodium_mean_ compared to those with favorable outcomes (27.8% vs. 8.4%, 135.9 ± 5.8 vs. 138.4 ± 4.3, and 139.1 ± 5.1 vs. 141.3 ± 3.9, all *p* < 0.001). Multivariate analysis revealed that hyponatremia was an independent predictor of adverse outcome (OR 3.579, 95%CI 2.332–6.780, p < 0.001), controlling for age, hemorrhage volume, IVH (Graeb score), admission GCS score, and ICH score.

**Table 4:** Comparisons of demographics between patients with favorable (mRS ≤ 3) and adverse outcomes (mRS > 3).

	Favorable	Adverse	Value of *p*
Patients	406	554	
Age (years)	64.6 ± 11.9	66.2 ± 12.6	0.047
Male, *n* (%)	266 (65.5)	338 (61.0)	0.153
BMI	24.9 (23.2–27.6)	25.1 (23.0–27.8)	0.869
Hypertension, *n* (%)	324 (79.8)	449 (81.0)	0.631
Diabetes mellitus, *n* (%)	80 (19.7)	135 (24.4)	0.087
Dyslipidemia, *n* (%)	46 (11.3)	72 (13.0)	0.437
Hypercholesterolemia, *n* (%)	114 (28.1)	139 (25.1)	0.299
Atrial fibrillation, *n* (%)	42 (10.3)	61 (11.0)	0.742
Previous stroke, *n* (%)	66 (16.3)	90 (16.2)	0.996
History of CHD, *n* (%)	16 (3.9)	14 (2.5)	0.622
History of renal insufficiency, *n* (%)	45 (11.1)	65 (11.7)	0.755
History of liver disease, *n* (%)	102 (25.1)	125 (22.6)	0.356
Hematoma volume (ml)	15 (8–31)	32 (18–40)	< 0.001
IVH, *n* (%)	193 (47.5)	334 (60.3)	< 0.001
Graeb score	0 (0–2)	1 (0–2)	< 0.001
Hematoma location, *n* (%)			0.144
Deep	338 (83.3)	480 (86.6)	
Lobar	68 (16.7)	74 (13.4)	
Admission GCS score	13 (8–15)	11 (8–14)	< 0.001
Admission ICH score	1 (1–2)	2 (1–3)	< 0.001
Hypernatremia, *n* (%)	69 (17.0)	81 (14.6)	0.317
Hyponatremia, *n* (%)	34 (8.4)	154 (27.8)	< 0.001
Sodium_min_	138.4 ± 4.3	135.9 ± 5.8	< 0.001
Sodium_mean_	141.3 ± 3.9	139.1 ± 5.1	< 0.001

BMI, body mass index; CHD, coronary heart disease; GCS, glasgow coma scale; ICH, intracerebral hemorrhage.

### 3.4 Complications in hospital

The common complications after sICH are presented in Table 5. However, patients with hyponatremia did not have an increased rate of complications compared to those without hyponatremia (all *p* > 0.05).

**Table 5:** Comparison of patients with and without hyponatremia.

	Hyponatremia	Non-hyponatremia	*p* value
Pneumonia	95 (50.5)	352 (45.6)	0.224
Meningitis	10 (5.3)	33 (4.3)	0.535
Sepsis	13 (6.9)	32 (4.1)	0.345
Urinary tract infection	23 (12.2)	81 (10.5)	0.491
Seizure	6 (3.2)	20 (2.6)	0.649
Hydrocephalus	17 (9.0)	65 (8.4)	0.784

## 4 Discussion

In the current study, we present a comprehensive analysis of early hyponatremia (first 7 days) in supratentorial sICH patients and reveal hyponatremia as an independent predictor of both 90-day mortality and adverse outcome. Moreover, subgroup analysis first identified the predictive value of hyponatremia for 90-day mortality in patients under different conditions.

The incidence of hyponatremia in our cohort was 19.6%, which is similar to the rate reported by Gray and colleagues in the same period after hemorrhage (15.2%) (15). In this study, we only analyzed the effect of hyponatremia within 7 days after bleeding to maintain consistency between cases. In addition, we also considered that the early stage (first 7 days) after ICH onset was of clinical importance in terms of the formation of the cerebral edema, the transformation of neuroinflammation, and the occurrence of early seizures, all of which were identified as associated with increased mortality (33–37). Therefore, we conducted this study to elucidate whether hyponatremia within 7 days after ICH is associated with 90-day outcomes.

Numerous research studies have been carried out on hyponatremia in traumatic brain injury, SAH, and neurosurgical intervention, confirming the association between hyponatremia and increased mortality (7–9, 17). However, much uncertainty remains regarding the relationship between decreased serum sodium and prognosis in patients with sICH. Table 6 features a summary of published studies on hyponatremia or serum sodium concentration in cases of ICH. Consistent with two comparatively large cohorts of ICH reported by Kuramatsu et al. (17) and Carcel et al. (20), we identified a 2.7-fold increase in 90-day mortality among patients with hyponatremia compared to normonatremia patients. However, contrary to our findings, Mansouri et al. (38) suggested that, through the comparison of 30-day mortality, a significantly lower sodium level was found in the survivor group. Moreover, no significant difference in sodium levels between survivor and non-survivor groups was identified when 3-month mortality was compared (38). However, limited sample size (*n* = 120) may impair the efficacy of their results. The mechanism linking hyponatremia and mortality is still undetermined. Kuramatsu et al. conducted the first study concerning the association between hyponatremia and mortality in a case series of ICH and considered that hyponatremia may serve as an indicator of disease severity, potentially reflecting a poor prognosis (17). A hypothesis underlying the mechanism of hyponatremia influencing outcome implicates its relationship with arginine vasopressin (AVP). Increased intracerebral pressure and cerebral hypoperfusion after ICH may aggravate secondary brain injury and elevate the nonosmotic release of AVP through the overstimulation of the neurohumoral axis (39, 40). Increased AVP levels themselves may result in a reduced tolerance to cerebral hypoxia (41). On the other hand, Tzoulis et al. reported a case–control study, suggesting that hyponatremia independently has adverse effects on the physiologic functions of the brain or other systems (42). Fatal brain edema due to severe hyponatremia and osmotic demyelination syndrome, a potentially fatal condition caused by overly rapid correction of hyponatremia, may contribute to increased mortality (42). Nevertheless, we did not confirm any fatalities due to these complications in this study. In our cohort, we attempted to explore the relationship between hyponatremia severity and duration with variables that univariately predicted 90-day mortality. However, only weak correlations were found with several factors, and no significant correlation was found in the non-survivor group. Therefore, we consider that hyponatremia certainly impairs the prognosis of ICH patients, while a single view is insufficient to explain the correlation between hyponatremia and increased mortality. Macroscopically, hyponatremia reflects the severity of ICH. For patients with mass hematoma or cerebral edema, high-dose osmotic agents and diuretics may be used to release excessive cerebrospinal fluid to reduce intracerebral pressure but also increase sodium excretion simultaneously (8, 9, 20). Even if individualized treatment has been adopted to keep the balance of body fluid in ICU, maintaining homeostasis of these patients is rather difficult, especially for neurosurgical patients who are more critical than general patients and are often complicated with aspiration pneumonia, acute renal failure, or other complications (8, 28). Microscopically, hyponatremia may decrease extracellular plasma osmolality, leading to osmotic swelling of brain cells, further increasing the risk of cerebral edema, seizures, and delayed cerebral infarctions (17). However, the above mechanism indicates that hyponatremia may indirectly promote mortality by causing other morbidity, compared to other direct variables, like hemorrhage volume and admission GCS score (15). This may explain the results depicted in the ROC curves that the diagnostic value of sodium_min_ for mortality is limited.

**Table 6:** Summary of literature on hyponatremia or sodium level of patients with ICH.

Authors and years	No. of patients	No. of hyponatremia	No. of mortality	Outcome measures	Results
Carcel et al. (20)	3,002	349 (11.6%)	353 (11.7%)	90-day mortality	Higher mortality in hyponatremia group.
Gray et al. (15)	99	24 (24.2%)	30 (30.3%)	In-hospital mortality	No significant difference in mortality between hyponatremia and non-hyponatremia groups
Koivunen et al. (16)	325	146 (44.9%)	55 (16.9%)	3-month mortality	No significant difference in hyponatremia incidence between survivor and non-survivor groups.
Kuramatsu et al. (17)	422	66 (15.6%)	102 (24.2%) in-hospital 138 (32.7%) at 90-day	In-hospital mortality 90-day mortality	and (2): Higher mortality in hyponatremia group.
Shah et al. (18)	234	106 (45.3%)	38 (16.2%)	In-hospital mortality	Higher mortality in hyponatremia group.
Li et al. (19)	227	NA	49 (21.6%)	In-hospital mortality	No significant difference in admission Na level between survivor and non-survivor groups.
Mansouri et al. (38)	120	NA	30 (25%) at 30-day 26 (28.9%) at 3-month	30-day mortality 3-month mortality	Significantly lower sodium level in survivor group. No significant difference in sodium levels between survivor and non-survivor groups

SIADH and CSW are considered to be the main causes of post-stroke hyponatremia (7). SIADH is developed by an excessive secretion of antidiuretic hormone (ADH) that is associated with water retention, and treatment is mainly based on water restriction. On the other hand, CSW may be generated by the release of brain natriuretic peptide (BNP) and atrial natriuretic peptide (ANP), both of which are associated with diuresis and natriuresis, resulting in hypovolemia, and treatment relies on supplemental sodium (40). Elevated intracerebral pressure (ICP) and neurologic injury may cause SIADH and CSW syndrome to disturb sodium homeostasis (9). An irritant effect on the hypothalamic–pituitary axis leading to the excessive release of ADH may result in SIADH (43). BNP increased in proportion to the increases in ICP, and CSW was believed to be the primary cause of hyponatremia in subarachnoid hemorrhage, acute encephalitis syndrome, and meningitis (44–46). Consistent with the findings of Gray et al. (15), we also observed that SIADH was the most common cause of hyponatremia. A contrasting finding was also reported by Kalita et al. (9), who put forward that CSW was the most common cause of hyponatremia in stroke. The discrepancy may be attributed to the fact that we only measured the hyponatremia within 7 days after hemorrhage, while hyponatremia occurred in their study after a median delay of 9.7 days from ictus. We hypothesize that the mass effect on the hypothalamic–pituitary axis caused by increased predominantly focal ICP after supratentorial sICH, like midline shift, could dominate SIADH as the main etiology of hyponatremia. On the other hand, Tripathi et al. put forward that hyponatremia due to CSW is associated with high catecholamine levels, which has also been reported in ICH and may correspond to the stress response (47, 48). This may explain the occurrence of CSW in patients with ICH. Nevertheless, caution needs to be exercised when interpreting these results because we can only analyze the etiology through medical records due to the insufficient awareness of hyponatremia after ICH at that time. Moreover, the cause of hyponatremia may not be easy to diagnose in a retrospective study. Additional studies with a multicenter prospective design may provide more robust data for the analysis of the etiology of hyponatremia after ICH.

Critically, there has been no evidence (direct or indirect) to prove whether correcting hyponatremia is appropriate after sICH (15–20). Some studies have suggested that impaired cerebral adaption mechanisms may result in demyelinizations at normal serum sodium levels after neurological injury/hypoxia, speculating that correction of hyponatremia could even be harmful (39, 49). In our study, an increased trend of mortality risk with degree of hyponatremia demonstrated the necessity of normal sodium maintenance and appropriateness of hyponatremia correction for patients with supratentorial sICH. In addition, we first explored the relationship between the duration of hyponatremia and mortality in ICH patients, finding that patients with hyponatremia lasting more than 48 h have a higher risk of mortality compared to normonatremic and short-term (less than 48 h) hyponatremic cohorts. The onset of acute hyponatremia occurs within 48 h, while chronic hyponatremia develops in over 48 h (50–52). Baker et al. (53) put forward that neurotoxic factors affect the brain through a leaky blood–brain barrier (BBB) to induce osmotic demyelination syndrome, while osmotic stress could more easily impair the barrier during chronic hyponatremia. Delayed treatment and accumulated risk of demyelination during sodium correction in chronic hyponatremia may be more likely to result in adverse outcomes compared to those in acute hyponatremia (50–52). Therefore, we recommend that rapid correction of hyponatremia is harmful because the BBB is damaged due to hemorrhage, and the nervous system may be more sensitive to fluctuation of serum sodium and higher risk of ton osmotic demyelination than the general.

In the subgroup analysis of treatment methods, we found that hyponatremia was significantly associated with an increased risk of mortality in patients with surgical intervention (EVD, craniotomy, and DC). However, a significant result could not be achieved in patients treated conservatively. Although therapeutic methods (conservative or surgical) for sICH are controversial, we still consider that hematoma evacuation relieves mass effect and prevents the impact of blood products into surrounding healthy brain parenchyma (13). However, patients treated surgically commonly have a larger volume of hemorrhage, causing higher severity of neurological injury due to hemorrhage itself or iatrogenic damage when removing hematoma in the deep brain structure compared to those with conservative treatment (27, 28). On the one hand, hyponatremia may reflect a more severe condition as an indicator. On the other hand, reduced serum sodium levels in the early period after bleeding may deteriorate the severely injured nervous system.

The advantage of our study is the comparatively large sample size, which allowed for the detection of the relationship between hyponatremia and mortality based on classification of sodium level and subgroup analysis in detail (15, 17). However, it also had limitations. First, potential bias may be introduced for the retrospective analysis. Second, these results needed to be interpreted with caution, particularly the association between hyponatremia and 90-day mRS score. Since most of the patients had functional deficiency after ICH and could not be scheduled for out-patient follow-up, approximately half of the patients were only interviewed by phone. This may influence the accuracy of the evaluation of functional results, which also hampered the performance of further subgroup analysis for functional outcomes. Third, the value of serum sodium was well measured during hospitalization, but a small number of patients, who were discharged within 7 days after ICH onset, may potentially develop hyponatremia. Meanwhile, some patients with particularly severe ICH may have died before hyponatremia occurred; both of these conditions may affect the accuracy of this study. Fourth, a limited number of cases in some subgroups may also influence the results of our study. Fifth, endocrine scrutiny was not routinely performed, and we cannot determine whether there was hyponatremia caused by hypocortisolemia or hypothyroidism. Finally, the results of this study merely show statistical correlation rather than revealing a causal relationship. Studies that aim to elucidate the mechanisms that link hyponatremia to mortality are required in the future.

## 5 Conclusion

Hyponatremia is a common electrolyte disturbance in patients with supratentorial sICH and serves as an independent predictor of 90-day mortality and adverse outcomes. Increased trend of mortality risk with degree and duration of hyponatremia may indicate the appropriateness of hyponatremia correction. Much attention should be paid to serum sodium monitoring in patients treated surgically. Future studies are essential to explore the mechanisms that link hyponatremia to outcome.

## Data availability statement

The raw data supporting the conclusions of this article will be made available by the authors, without undue reservation.

## Ethics statement

The studies involving humans were approved by the Ethics Committee of Suining Central hospital. The studies were conducted in accordance with the local legislation and institutional requirements. Written informed consent for participation was not required from the participants or the participants’ legal guardians/next of kin in accordance with the national legislation and institutional requirements.

## Author contributions

AQ: Data curation, Writing – original draft. LZ: Formal analysis, Methodology, Visualization, Writing – review & editing. ZH: Validation, Visualization, Writing – review & editing. JZ: Formal analysis, Visualization, Writing – review & editing. ST: Conceptualization, Investigation, Supervision, Validation, Writing – review & editing. WX: Conceptualization, Investigation, Supervision, Validation, Writing – review & editing.

## References

[1] Kovesdy CP Lott EH Lu JL Malakauskas SM Ma JZ Molnar MZ. Hyponatremia, hypernatremia, and mortality in patients with chronic kidney disease with and without congestive heart failure. Circulation. (2012) 125:677–84. doi: 10.1161/CIRCULATIONAHA.111.065391, PMID: 22223429 PMC3294276

[2] Funk GC Lindner G Druml W Metnitz B Schwarz C Bauer P. Incidence and prognosis of dysnatremias present on ICU admission. Intensive Care Med. (2010) 36:304–11. doi: 10.1007/s00134-009-1692-0, PMID: 19847398

[3] Wald R Jaber BL Price LL Upadhyay A Madias NE. Impact of hospital-associated hyponatremia on selected outcomes. Arch Intern Med. (2010) 170:294–302. doi: 10.1001/archinternmed.2009.513, PMID: 20142578

[4] Klein L O'Connor CM Leimberger JD Gattis-Stough W Piña IL Felker GM. Lower serum sodium is associated with increased short-term mortality in hospitalized patients with worsening heart failure: results from the outcomes of a prospective trial of intravenous milrinone for exacerbations of chronic heart failure (OPTIME-CHF) study. Circulation. (2005) 111:2454–60. doi: 10.1161/01.CIR.0000165065.82609.3D, PMID: 15867182

[5] Rahman M Friedman WA. Hyponatremia in neurosurgical patients: clinical guidelines development. Neurosurgery. (2009) 65:925–36. doi: 10.1227/01.NEU.0000358954.62182.B3 19834406

[6] Moro N Katayama Y Igarashi T Mori T Kawamata T Kojima J. Hyponatremia in patients with traumatic brain injury: incidence, mechanism, and response to sodium supplementation or retention therapy with hydrocortisone. Surg Neurol. (2007) 68:387–93. doi: 10.1016/j.surneu.2006.11.052 17905062

[7] Ehtesham M Mohmand M Raj K Hussain T Kavita F Kumar B. Clinical Spectrum of hyponatremia in patients with stroke. Cureus. (2019) 11:e5310. doi: 10.7759/cureus.5310, PMID: 31592365 PMC6773452

[8] Saleem S Yousuf I Gul A Gupta S Verma S. Hyponatremia in stroke. Ann Indian Acad Neurol. (2014) 17:55–7. doi: 10.4103/0972-2327.128554, PMID: 24753660 PMC3992770

[9] Kalita J Singh RK Misra UK. Cerebral salt wasting is the Most common cause of hyponatremia in stroke. J Stroke Cerebrovasc Dis. (2017) 26:1026–32. doi: 10.1016/j.jstrokecerebrovasdis.2016.12.011, PMID: 28110888

[10] Specogna AV Turin TC Patten SB Hill MD. Factors associated with early deterioration after spontaneous intracerebral hemorrhage: a systematic review and meta-analysis. PLoS One. (2014) 9:e96743. doi: 10.1371/journal.pone.0096743, PMID: 24809990 PMC4014549

[11] Wang S Xu X Yu Q Hu H Han C Wang R. Combining modified Graeb score and intracerebral hemorrhage score to predict poor outcome in patients with spontaneous intracerebral hemorrhage undergoing surgical treatment. Front Neurol. (2022) 13:915370. doi: 10.3389/fneur.2022.915370, PMID: 35968295 PMC9373905

[12] Boland T Henderson GV Gibbons FK Brouwers HB Greenberg SM Raffeld M. Hypernatremia at hospital discharge and out of hospital mortality following primary intracerebral Hemorrhage. Neurocrit Care. (2016) 25:110–6. doi: 10.1007/s12028-015-0234-6, PMID: 26842718

[13] de Oliveira Manoel AL. Surgery for spontaneous intracerebral hemorrhage. Crit Care. (2020) 24:45. doi: 10.1186/s13054-020-2749-2, PMID: 32033578 PMC7006102

[14] Yan F Yi Z Hua Y Shen Y Li M Ding Y. Predictors of mortality and recurrent stroke within five years of intracerebral hemorrhage. Neurol Res. (2018) 40:466–72. doi: 10.1080/01616412.2018.1451266, PMID: 30134784

[15] Gray JR Morbitzer KA Liu-DeRyke X Parker D Zimmerman LH Rhoney DH. Hyponatremia in patients with spontaneous intracerebral Hemorrhage. J Clin Med. (2014) 3:1322–32., PMID: 26237605 10.3390/jcm3041322 PMC4470185

[16] Koivunen RJ Haapaniemi E Satopää J Niemelä M Tatlisumak T Putaala J. Medical acute complications of intracerebral hemorrhage in young adults. Stroke Res Treat. (2015) 2015:357696., PMID: 25722917 10.1155/2015/357696 PMC4333279

[17] Kuramatsu JB Bobinger T Volbers B Staykov D Lücking H Kloska SP. Hyponatremia is an independent predictor of in-hospital mortality in spontaneous intracerebral hemorrhage. Stroke. (2014) 45:1285–91. doi: 10.1161/STROKEAHA.113.004136, PMID: 24713532

[18] Shah A Sabir S Artani M Salam O Khan S Rizwan A. Significance of hyponatremia as an independent factor in predicting short-term mortality in patients with hemorrhagic stroke. Cureus. (2019) 11:e4549., PMID: 31275773 10.7759/cureus.4549 PMC6592831

[19] Li YF Luo J Li Q Jing YJ Wang RY Li RS. A new simple model for prediction of hospital mortality in patients with intracerebral hemorrhage. CNS Neurosci Ther. (2012) 18:482–6., PMID: 22672301 10.1111/j.1755-5949.2012.00320.x PMC6493661

[20] Carcel C Sato S Zheng D Heeley E Arima H Yang J. Prognostic significance of hyponatremia in acute intracerebral Hemorrhage: pooled analysis of the intensive blood pressure reduction in acute cerebral Hemorrhage trial studies. Crit Care Med. (2016) 44:1388–94. doi: 10.1097/CCM.0000000000001628, PMID: 26958746

[21] Morotti A Goldstein JN. Diagnosis and Management of Acute Intracerebral Hemorrhage. Emerg Med Clin North Am. (2016) 34:883–99. doi: 10.1016/j.emc.2016.06.010, PMID: 27741993 PMC5089075

[22] Kothari RU Brott T Broderick JP Barsan WG Sauerbeck LR Zuccarello M. The ABCs of measuring intracerebral hemorrhage volumes. Stroke. (1996) 27:1304–5. doi: 10.1161/01.STR.27.8.1304, PMID: 8711791

[23] Burgess RE Warach S Schaewe TJ Copenhaver BR Alger JR Vespa P. Development and validation of a simple conversion model for comparison of intracerebral hemorrhage volumes measured on CT and gradient recalled echo MRI. Stroke. (2008) 39:2017–20. doi: 10.1161/STROKEAHA.107.505719, PMID: 18483414 PMC2756493

[24] Hütter BO Kreitschmann-Andermahr I Gilsbach JM. Cognitive deficits in the acute stage after subarachnoid hemorrhage. Neurosurgery. (1998) 43:1054–64. doi: 10.1097/00006123-199811000-00030, PMID: 9802849

[25] Qian A Zhou J Yu J Huo G Wang X. Incidence and risk factors of delayed postoperative hyponatremia after endoscopic endonasal surgery for Rathke's cleft cyst: a single-center study. Front Surg. (2022) 9:953802. doi: 10.3389/fsurg.2022.953802, PMID: 35910473 PMC9334746

[26] Cui H He G Yang S Lv Y Jiang Z Gang X. Inappropriate antidiuretic hormone secretion and cerebral salt-wasting syndromes in neurological patients. Front Neurosci. (2019) 13:1170. doi: 10.3389/fnins.2019.01170, PMID: 31780881 PMC6857451

[27] Wang W Zhou N Wang C. Minimally invasive surgery for patients with hypertensive intracerebral Hemorrhage with large hematoma volume: a retrospective study. World Neurosurg. (2017) 105:348–58. doi: 10.1016/j.wneu.2017.05.158, PMID: 28602881

[28] Ma L Liu WG Sheng HS Fan J Hu WW Chen JS. Decompressive craniectomy in addition to hematoma evacuation improves mortality of patients with spontaneous basal ganglia hemorrhage. J Stroke Cerebrovasc Dis. (2010) 19:294–8. doi: 10.1016/j.jstrokecerebrovasdis.2009.07.002, PMID: 20452786

[29] Di Rienzo A Colasanti R Esposito D Della Costanza M Carrassi E Capece M. Endoscope-assisted microsurgical evacuation versus external ventricular drainage for the treatment of cast intraventricular hemorrhage: results of a comparative series. Neurosurg Rev. (2020) 43:695–708., PMID: 31069562 10.1007/s10143-019-01110-7

[30] Kim DB Park SK Moon BH Cho BR Jang DK Jang KS. Comparison of craniotomy and decompressive craniectomy in large supratentorial intracerebral hemorrhage. J Clin Neurosci. (2018) 50:208–13. doi: 10.1016/j.jocn.2018.01.066, PMID: 29428269

[31] Wang WJ Lu JJ Wang YJ Wang CX Wang YL Hoff K. Clinical characteristics, management, and functional outcomes in Chinese patients within the first year after intracerebral hemorrhage: analysis from China National Stroke Registry. CNS Neurosci Ther. (2012) 18:773–80. doi: 10.1111/j.1755-5949.2012.00367.x, PMID: 22943144 PMC6493640

[32] Ren Y Zheng J Liu X Li H You C. Risk factors of Rehemorrhage in postoperative patients with spontaneous intracerebral Hemorrhage: a case-control study. J Korean Neurosurg Soc. (2018) 61:35–41. doi: 10.3340/jkns.2017.0199, PMID: 29354234 PMC5769850

[33] Wan Y Holste KG Hua Y Keep RF Xi G. Brain edema formation and therapy after intracerebral hemorrhage. Neurobiol Dis. (2023) 176:105948. doi: 10.1016/j.nbd.2022.105948, PMID: 36481437 PMC10013956

[34] Tschoe C Bushnell CD Duncan PW Alexander-Miller MA Wolfe SQ. Neuroinflammation after intracerebral Hemorrhage and potential therapeutic targets. J Stroke. (2020) 22:29–46. doi: 10.5853/jos.2019.02236, PMID: 32027790 PMC7005353

[35] Feleppa M Di Iorio W Saracino DM. Early poststroke seizures. Clin Exp Hypertens. (2006) 28:265–70. doi: 10.1080/10641960600549215 16833033

[36] De Herdt V Dumont F Hénon H Derambure P Vonck K Leys D. Early seizures in intracerebral hemorrhage: incidence, associated factors, and outcome. Neurology. (2011) 77:1794–800. 21975203 10.1212/WNL.0b013e31823648a6

[37] Guo X Xu JK Qi X Wei Y Wang CW Li H. Early brainstem hemorrhage progression: multi-sequence magnetic resonance imaging and histopathology. Neural Regen Res. (2023) 18:170–5. doi: 10.4103/1673-5374.344838, PMID: 35799538 PMC9241409

[38] Mansouri B Heidari K Asadollahi S Nazari M Assarzadegan F Amini A. Mortality and functional disability after spontaneous intracranial hemorrhage: the predictive impact of overall admission factors. Neurol Sci. (2013) 34:1933–9. doi: 10.1007/s10072-013-1410-0, PMID: 23543380

[39] Schrier RW Sharma S Shchekochikhin D. Hyponatraemia: more than just a marker of disease severity? Nat Rev Nephrol. (2013) 9:37–50. doi: 10.1038/nrneph.2012.246, PMID: 23165296

[40] Brimioulle S Orellana-Jimenez C Aminian A Vincent JL. Hyponatremia in neurological patients: cerebral salt wasting versus inappropriate antidiuretic hormone secretion. Intensive Care Med. (2008) 34:125–31. doi: 10.1007/s00134-007-0905-7, PMID: 17952405

[41] Wallace BK Foroutan S O'Donnell ME. Ischemia-induced stimulation of Na-K-cl cotransport in cerebral microvascular endothelial cells involves AMP kinase. Am J Physiol Cell Physiol. (2011) 301:C316–26. doi: 10.1152/ajpcell.00517.2010, PMID: 21562306 PMC3154550

[42] Tzoulis P Bagkeris E Bouloux PM. A case-control study of hyponatraemia as an independent risk factor for inpatient mortality. Clin Endocrinol. (2014) 81:401–7. doi: 10.1111/cen.12429, PMID: 24612060

[43] Mapa B Taylor BE Appelboom G Bruce M Claassen J Connolly ES Jr.. Impact of hyponatremia on morbidity, mortality, and complications after aneurysmal subarachnoid Hemorrhage: a systematic review. World Neurosurg. (2016) 85:305–14., PMID: 26361321 10.1016/j.wneu.2015.08.054

[44] Misra UK Kalita J Singh RK Bhoi SK. A study of hyponatremia in acute encephalitis syndrome: a prospective study from a tertiary care Center in India. J Intensive Care Med. (2019) 34:411–7. doi: 10.1177/0885066617701422, PMID: 28393593

[45] Misra UK Kalita J. Mechanism, spectrum, consequences and management of hyponatremia in tuberculous meningitis. Wellcome Open Res. (2019) 4:189. doi: 10.12688/wellcomeopenres.15502.1 32734004 PMC7372311

[46] Berendes E Walter M Cullen P Prien T Aken HV Horsthemke J. Secretion of brain natriuretic peptide in patients with aneurysmal subarachnoid haemorrhage. Lancet. (1997) 349:245–9. doi: 10.1016/S0140-6736(96)08093-2 9014912

[47] Tripathi A Thakur RS Kalita J Patel DK Misra UK. Is cerebral salt wasting related to sympathetic dysregulation in tuberculous meningitis? Neurosci Lett. (2021) 747:135671. doi: 10.1016/j.neulet.2021.135671, PMID: 33516801

[48] Meyer JS Stoica E Pascu I Shimazu K Hartmann A. Catecholamine concentrations in CSF and plasma of patients with cerebral infarction and haemorrhage. Brain. (1973) 96:277–88., PMID: 4715186 10.1093/brain/96.2.277

[49] Hoorn EJ Zietse R. Hyponatremia and mortality: moving beyond associations. Am J Kidney Dis. (2013) 62:139–49. doi: 10.1053/j.ajkd.2012.09.019, PMID: 23291150

[50] Decaux G Soupart A. Treatment of symptomatic hyponatremia. Am J Med Sci. (2003) 326:25–30. doi: 10.1097/00000441-200307000-00004 12861122

[51] Sahay M Sahay R. Hyponatremia: a practical approach. Indian J Endocrinol Metab. (2014) 18:760–71. doi: 10.4103/2230-8210.141320, PMID: 25364669 PMC4192979

[52] Sterns RH. Adverse consequences of overly-rapid correction of hyponatremia. Front Horm Res. (2019) 52:130–42. doi: 10.1159/000493243, PMID: 32097948

[53] Baker EA Tian Y Adler S Verbalis JG. Blood-brain barrier disruption and complement activation in the brain following rapid correction of chronic hyponatremia. Exp Neurol. (2000) 165:221–30. doi: 10.1006/exnr.2000.7474, PMID: 10993682

